# Occurrence and Dietary Risk Assessment of *Alternaria* Toxin in Edible Mushrooms: An Application of a Hydrophilic Solid-Phase Microextraction Fiber

**DOI:** 10.3390/foods15111992

**Published:** 2026-06-03

**Authors:** Zhenqin Zhao, Lu Sun, Jiaqi Liu, Dali Sun, Chaoxuan Liao, Yan Man, Zelan Wang, Shuang Lei, Qinghai Zhang, Zhoubing Huang

**Affiliations:** 1 The Key Laboratory of Environmental Pollution Monitoring and Disease Control, Ministry of Education, School of Public Health, Guizhou Medical University, Guian New Area, Guiyang 561113, China; 2 Guizhou Academy of Testing and Analysis, Guiyang 550000, China; liaochaoxuan@gzata.cn; 3 Institute of Quality Standard and Testing Technology, Beijing Academy of Agriculture and Forestry Sciences, Beijing 100097, China

**Keywords:** *Alternaria* mycotoxins, solid-phase microextraction, mushroom safety, dietary exposure

## Abstract

Edible mushrooms are widely consumed across the globe. However, the critical lack of reliable detection methods has led to severely limited data on the contamination profiles and dietary exposure risks of *Alternaria* toxins in edible mushrooms. This study utilized a hydrophilic–lipophilic balanced solid-phase microextraction fiber for the detection of *Alternaria* toxins in edible mushrooms. The employed VIM/DVB fiber exhibited superior performance with a wide linear range (1–200 μg/kg), low limits of detection (0.01–0.26 μg/kg) and satisfactory recoveries (80.6–111.6%). Applying this method to 63 edible mushrooms (6 species) from Guiyang (Guizhou, China) revealed varying degrees of contamination, with all samples containing at least one of four target toxins (alternariol, alternariol monomethyl ether, altenuene, tentoxin). Tentoxin (1.86 ± 2.6 μg/kg, 76.2% detection frequency) and alternariol (1.61 ± 1.24 μg/kg, 55.6% detection frequency) were the predominant toxins. Toxin distribution varied by mushroom species. *Lentinula edodes* was prone to tentoxin contamination, while *Pleurotus eryngii* was even more susceptible to alternariol-tentoxin co-occurrence. Dietary risk assessment indicated that alternariol posed the highest risks, with 51% samples exceeding the toxicological threshold (2.5 ng/kg bw/day). This study provides a reliable detection tool for *Alternaria* toxins in edible mushrooms and highlights the signal that continuous attention needs to be paid to *Alternaria* toxins in edible mushrooms.

## 1. Introduction

The global edible mushroom industry has been expanding steadily in recent years. It is expected that the global annual production will reach 20.84 million tons by 2026 [1]. As a key producer of edible mushrooms, China yielded 42.2254 million tons in 2022, generating a total value of approximately 388.7 billion yuan. The edible mushrooms has become the fifth largest agricultural sector in China, trailing grain, vegetables, fruits, and oilseeds [2,3]. Nevertheless, although commercial edible mushrooms are currently not commonly contaminated by *Alternaria*, the high-moisture and nutrient-rich cultivation conditions make mushrooms particularly vulnerable to infection by *Alternaria*, posing a potential risk to agricultural product safety [4]. For example, in Huaihua, China, isolated Alternaria alternata can trigger apothecium malformation of cultivated Morchella importuna. *Trichoderma* exhibits low toxicity and mainly causes economic losses. By contrast, the optimal water activity (a_w_ < 0.85) for toxin production by *Aspergillus* is poorly matched with the high-humidity cultivation environment of edible mushrooms (a_w_ > 0.95). Despite its sporadic occurrence and low infection rate (5–10%) under conventional cultivation, *Alternaria* can damage fruiting bodies and generate over 70 Alternaria mycotoxins [5], which necessitates ongoing monitoring.

*Alternaria* toxins are a class of toxic metabolites produced by fungi of the genus *Alternaria* [6,7,8,9]. Some *Alternaria* toxins, such as alternariol (AOH), alternariol monomethyl ether (AME), and tenuazonic acid (TeA), have been demonstrated to exhibit various health risks, including genotoxicity, carcinogenicity, and embryotoxicity [4,10,11]. Notably, AOH and AME can directly cause DNA damage by inhibiting DNA topoisomerases [12,13], with AME being a potent dual inhibitor of both topoisomerase I and II, highlighting its significant genetic virulence [14]. The European Food Safety Authority (EFSA) has set safety cutoff values following the toxicological concern framework, assigning a reference dose of 2.5 ng/kg bw/day for both AOH and AME [10]. Current monitoring indicates that dietary exposure levels in certain populations are approaching or exceeding these limits, suggesting that the associated public health risks cannot be ignored [15].

Recent research and monitoring of *Alternaria* toxins primarily focus on staple agricultural products, including cereals, vegetables, and fruits. There is a significant lack of attention and data regarding edible mushrooms, despite their high consumption volume, making it difficult to accurately assess their dietary exposure risks. Existing mainstream sample preparation methods, such as liquid–liquid extraction [16], solid-phase extraction (SPE) [17], and QuEChERS [18,19], have inherent limitations. These include low recovery rates (e.g., acetonitrile/methanol, only 48–76% recovery for AOH, TeA, and AME in aqueous conditions) [20], high limits of detection (e.g., QuEChERS exhibits an LOD as high as 27.8 ng/g for TeA), and excessive organic solvent usage [21,22]. Additionally, complex polysaccharides in edible mushrooms, such as chitin and β-glucan, easily interact with highly polar *Alternaria* toxins, further interfering with extraction efficiency [23]. Thus, there is an urgent need to employ novel, targeted detection technologies.

Solid-phase microextraction (SPME) is a low-solvent, highly efficient concentration characterized by its simplicity, minimal sample requirements, and high enrichment efficiency [24]. Compared to traditional methods, SPME enables targeted enrichment through specific interactions between the fiber coating and the target analytes, effectively reducing matrix interference [25]. Additionally, it eliminates the need for large volumes of organic solvents, thereby avoiding secondary interactions between solvents and polysaccharide matrices inherent in conventional methods [26].

Therefore, this study aims to employ a hydrophilic–lipophilic balanced SPME fiber. Through optimization and systematic evaluation of its analytical performance, an efficient detection protocol for *Alternaria* toxins in edible mushrooms was established. Based on this technology, 63 edible mushrooms (covering six species) from Guiyang were analyzed to reveal their contamination characteristics and distribution patterns, as well as to conduct a preliminary assessment of dietary exposure risks. This research not only provides a reliable technical solution for the accurate quantification of *Alternaria* toxins in edible mushrooms but also establishes a critical scientific basis and data support for formulating future limit standards and improving risk management strategies by clarifying the current contamination status and risk levels.

## 2. Materials and Methods

### 2.1. Chemicals and Materials

The standards of the four *Alternaria* toxins, including alternariol (AOH), alternariol monomethyl ether (AME), altenuene (ALT) and tentoxin (TEN), were procured from Qingdao Pribolab Biotech Co., Ltd. (Qingdao, China). Divinylbenzene (80% purity, DVB), 1-vinylimidazole (98% purity, VIM), 2,2-azobisisobutyronitrile (AIBN), methanol, acetone and acetonitrile were purchased from Shanghai Aladdin Biochemical Technology Co., Ltd. (Shanghai, China). Analytically pure ammonium acetate was obtained from Chengdu Kelong Chemical Reagent Factory. Stainless steel (SS) wire (150 μm diameter, medical grade) was purchased from Small Parts (Miami Lakes, FL, USA). HLB cartridges (200 mg, 60 μm) were bought from Ningbo Hongpu Experimental Technology Co., Ltd. (Ningbo, China).

### 2.2. Sample Collection

A total of 63 fresh edible mushrooms were collected in spring 2025 (March) from 14 major agricultural markets in Guiyang, the capital city of Guizhou province, China. These samples comprised 6 different species, consisting of 7 *Agaricus bisporus*, 9 *Hypsizygus marmoreus*, 9 *Flammulina velutipes*, 12 *Pleurotus ostreatus*, 14 *Lentinula edodes*, and 12 *Pleurotus eryngii*. During sampling, all collected specimens were placed in sterile, labeled polyethylene bags, kept in an ice box, and transported to the laboratory within 4 h. Each sample was collected from independent batches to avoid cross-contamination. All sampling tools were pre-cleaned with 75% ethanol and ultrapure water before use. In addition, we examined the morphological characteristics of each sample and compared representative specimens with standard reference materials and the literature to verify the primary species. We test the edible portions of mushroom fruiting bodies. These included a mixture of caps, gills, and stems, as is typical for common consumption of these edible mushrooms. Before homogenization, inedible parts, such as the stems and debris adhering to the growth medium, are removed. All edible tissue is thoroughly mixed and homogenized to create a composite sample, ensuring that the measured concentrations reflect the entire mushroom as it would be consumed.

### 2.3. Preparation of SPME Fiber

The preparation of solid-phase microextraction fibers was conducted based on the preliminary work of the reference laboratory [27]. Stainless steel wires (150 μm) were sectioned into 4 cm segments. Sequentially, the wire segments underwent ultrasonic cleaning in water, methanol and acetone to remove surface contaminants. The cleaned wires were air-dried prior to subsequent processing. In a 1.5 mL centrifuge tube, 0.94 mL of VIM and 0.21 mL of DVB were precisely measured, followed by addition of 0.03 g AIBN as initiator. The mixture was homogenized with vigorous shaking for 2 min. This precursor solution was then loaded into 0.5 mm diameter glass capillary tubes, ensuring complete filling without air bubble formation. A pre-treated stainless steel wire was subsequently inserted into the filled capillary. The assembly was horizontally oriented in a Petri dish and polymerized in a forced-air oven at 60 °C for 6 h. After polymerization, the coated fiber was carefully excised using a surgical blade, preserving 1.0 cm active coating segments at both termini. Finally, the polymeric coating with a diameter of 175 μm was extracted with methanol (5 mL) to remove residual monomers and impurities.

### 2.4. Instrumental Analysis

An Agilent triple quadrupole mass spectrometer (G6475A) coupled with high-performance liquid chromatography was operated in electrospray ionization (ESI) mode. Chromatographic separation was carried out on an InfinityLab Poroshell 120 EC-C18 column (3.0 × 50 mm, 2.7 μm) under a constant column temperature of 40 °C. The sample injection volume was fixed at 5 μL, and the mobile phase was delivered at a flow rate of 0.3 mL/min. The mobile phase consisted of 1 mM ammonium acetate aqueous solution (Phase A) and pure methanol (Phase B). The gradient elution procedure was designed as follows: 90% Phase A from 0 to 0.5 min; a linear decrease from 90% to 10% Phase A during 0.5–3.0 min; maintained at 10% Phase A for 3.0–3.5 min; then returned linearly to 90% Phase A within 3.5–4.0 min, and finally kept at 90% Phase A until the end of the 5.0 min run. Other mass spectrometric parameters for the four target analytes are presented in Table S1. This study did not analyze TeA, as this compound readily chelates with metal ions (e.g., Fe^2+^ and Cu^2+^) to form stable complexes, which impairs its mass spectrometric response and quantitative stability [28].

### 2.5. Sample Pre-Treatment of Edible Mushrooms

SPE method: Precisely weighed edible mushrooms (1.00 ± 0.01 g) were placed into 50 mL polypropylene centrifuge tubes. Each sample was extracted using 5 mL of acidified acetonitrile/water (84:16, *v*/*v*, 1.0% formic acid) via ultrasonication at ambient temperature for 30 min. After centrifugation at 8000 rpm for 20 min to complete phase partitioning, the supernatant was transferred into a 15 mL tube. The extraction operation was conducted twice in total, and all collected supernatants were combined and concentrated to nearly dryness by nitrogen blowing at 40 °C. The dried residue was redissolved in 1.0 mL of ultrapure water for subsequent purification. Before sample loading, HLB SPE cartridges (200 mg/6 mL) were pre-conditioned sequentially with 6 mL methanol and 6 mL deionized water at a constant flow rate of 1 mL/min. Subsequently, 10 mL of methanol was employed as the eluent, and the resulting eluate was collected. Finally, the methanol eluate was concentrated to a final volume of 1 mL and filtered through a 0.22 μm PTFE membrane before HPLC-MS/MS instrumental determination.

SPME Method: Fresh edible mushrooms (10.0 ± 0.1 g) were homogenized with ultrapure water (1:1, *w*/*w*) using a high-speed tissue disruptor. After homogenization, the sample solution was centrifuged at 8000× *g* for 15 min, and the resulting supernatant was filtered using a 0.45 μm PTFE membrane. Exactly 10 mL of the filtrate was taken and poured into a 10 mL glass vial. Afterwards, the prepared SPME fiber was immersed in the liquid sample to perform adsorption for 60 min. Subsequently, the fiber was withdrawn and immersed in 200 μL of methanol for desorption for 40 min. Finally, the fiber was removed, and the methanol desorption solution was analyzed via HPLC-MS/MS (Figure S1).

### 2.6. Method Development and Validation

The analytical performance of the established SPME-HPLC-MS/MS approach was assessed in terms of linearity range, limit of detection (LOD), limit of quantification (LOQ), extraction recovery and methodological precision. To compensate for matrix-induced signal suppression or enhancement, matrix-matched calibration curves were constructed for the accurate quantification of *Alternaria* toxins in edible mushrooms.

Matrix-matched standard solutions of *Alternaria* toxins, ranging from 1 to 200 μg/kg, were prepared using blank *Pleurotus ostreatus* samples. For each concentration level, the peak areas of the four *Alternaria* toxins were measured in triplicate. Calibration curves were established by plotting peak area on the vertical axis against analyte mass concentration (μg/kg) on the horizontal axis. Linear regression analysis was adopted to calculate the slope, intercept and correlation coefficient of each standard curve. The LOD and LOQ values were determined according to signal-to-noise ratios of 3:1 and 10:1 for target compounds, respectively. Method validation also included recovery experiments, which were conducted by spiking *Alternaria* toxin-free *Pleurotus ostreatus* matrices at three concentration levels: 10 μg/kg (low), 50 μg/kg (medium), and 200 μg/kg (high). Each fortification level was prepared independently in triplicate (*n* = 3) and analyzed under the optimized chromatographic conditions to evaluate the accuracy of the method.

Group differences were analyzed using one-way ANOVA. The Shapiro–Wilk test and Levene’s test were separately adopted to assess the normality of distribution and homogeneity of variance. One-way analysis of variance was applied when the above prerequisites were satisfied; otherwise, the nonparametric Kruskal–Wallis test was selected for statistical comparison. The Bonferroni correction method was utilized to eliminate the type I error in multiple pairwise comparisons.

### 2.7. Dietary Exposure Risk Assessment of Alternaria Toxins in Edible Mushrooms

The dietary risk of *Alternaria* toxins in edible mushrooms was assessed using a distribution-based (percentile) exposure approach, which involved constructing exposure rank distributions (ERDs) and calculating hazard quotients (HQs). The concentrations of *Alternaria* toxins in edible mushrooms were arranged in ascending order, and percentiles were assigned using the Weibull formula (Equation (1)):

(1) $$\mathrm{Percentile}\;(\%)=\frac{i\;\times \;100}{n\;+\;1}(\%)$$

The dietary exposure (Exp) to *Alternaria* toxins through the consumption of edible mushrooms by different populations in selected districts of Guiyang was calculated using Equation (2).

(2) $$\mathrm{Exp}=C\;\times \;\frac{F}{\mathrm{BW}}$$

where Exp represents the dietary exposure to *Alternaria* toxins (ng/kg·bw/d); C denotes the concentration of *Alternaria* toxins in edible mushrooms (μg/kg); BW corresponds to the average adult body weight (59.10 kg) [29]; and F is the daily consumption rate of edible mushrooms (122.5 g/d for adults) [30].

The hazard quotient (HQ) was calculated as the ratio of the estimated daily intake (EDI) to the threshold of toxicological concern (TTC) (Equation (3)). An HQ < 1.0 indicates acceptable exposure, whereas an HQ > 1.0 indicates a potential health risk [31,32]. The HQ was calculated using the following equation:

(3) $$\mathrm{HQ}=\;\frac{\mathrm{Exp}}{\mathrm{TTC}}$$

Here, the TTC values established by the European Food Safety Authority (EFSA) for *Alternaria* toxins were 2.5 ng/kg bw/d for AOH and AME, and 1500 ng/kg bw/d for TEN [10]. Accordingly, a TTC value of 2.5 ng/kg bw/d was adopted for ALT [33].

## 3. Results and Discussion

### 3.1. Preparation and Characterization of Solid-Phase Microextraction Fibers

The Fourier-transform infrared (FTIR) spectra of the SPME fibers are depicted in Figure S2. The absorption peaks observed at 1504, 1451, and 1416 cm^−1^ correspond to the stretching vibrations of the benzene ring in DVB. The absorption peaks at 1283, 1112, and 1083 cm^−1^ are attributed to the C-N stretching vibrations in VIM. Additionally, the absorption peak at 2931 cm^−1^ originates from the -CH_2_ stretching vibration, while the peak at 1642 cm^−1^ corresponds to the C=C stretching vibration [27]. The N_2_ adsorption–desorption isotherms and pore size distributions of the SPME fibers are presented in Figure S3 and Table S2, respectively. The specific surface area and pore volume of VIM/DVB were 121.3 m^2^/g and 0.43 cm^3^/g, respectively, which were significantly higher than those of DVB (4.6 m^2^/g and 0.008 cm^3^/g).

**Figure 1:**
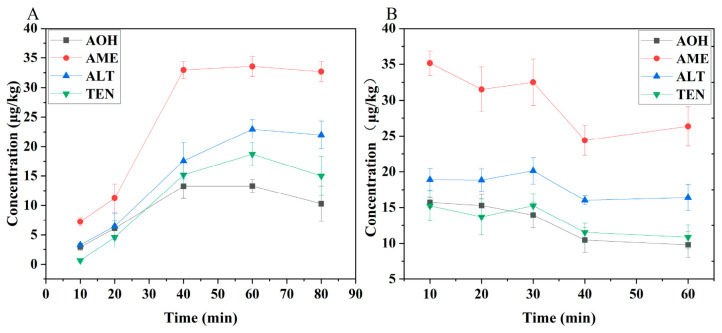
Effect of adsorption time (**A**) and desorption time (**B**) on extraction efficiency. AOH: alternariol; AME: alternariol monomethyl ether; ALT: altenuene; TEN: tentoxin. All experiments were performed at room temperature (25 °C), and no stirring was applied during the extraction process.

The adsorption of *Alternaria* toxins on the SPME fiber increased over time until equilibrium was reached. To achieve optimal extraction efficiency, both the adsorption and desorption durations required optimization. The effect of adsorption time (10–80 min) on extraction efficiency was therefore investigated. As shown in Figure 1A, analyte concentrations increased progressively from 10 to 40 min of adsorption and then plateaued. Notably, AOH and TEN exhibited less pronounced declines after reaching their maximum adsorption at 60 min, potentially due to competitive adsorption effects in the complex matrix. Since all compounds achieved optimal adsorption within 60 min, this duration was selected for subsequent desorption experiments. The effect of desorption time on extraction efficiency was investigated under the pre-established adsorption equilibrium condition of 60 min. As shown in Figure 1B, the concentrations of the desorbed compounds exhibited a decreasing trend from 10 to 30 min and gradually reached equilibrium after 40 min. This phenomenon may be attributed to the release of additional matrix interferences with prolonged desorption, which could compete with the target analytes for ionization efficiency in the mass spectrometer, thereby reducing the signal intensity. To ensure more stable detection results, a desorption time of 40 min was selected as optimal.

### 3.2. Method Validation

To evaluate the analytical performance of the developed method, control samples of *Pleurotus ostreatus* were spiked at seven concentration levels (2, 5, 10, 20, 50, 100, and 200 μg/kg) to construct calibration curves. As summarized in Table S3, the method exhibited a wide linear range (1–200 μg/kg) with excellent linearity for all analytes, achieving correlation coefficients (R^2^) ranging from 0.997 to 0.999. The limits of detection (LODs) and quantification (LOQs) were in the range of 0.01–0.26 μg/kg and 0.05–0.86 μg/kg, respectively. These results underscore a substantial enhancement in sensitivity compared to previously reported SPME methods for *Alternaria* toxins, which reported LODs as high as 25 μg/kg in cornflakes and 70 μg/kg in ice wine [34,35]. Table 1 summarizes the reported QuEChERS, SPE, and liquid–liquid microextraction methods. In general, this method exhibits higher sensitivity than QuEChERS, SPE and liquid–liquid microextraction methods. Additionally, the batch processing capability and minimal solvent requirements of SPME give it a distinct advantage in sample preparation for edible mushrooms. Although direct comparative analysis with natural or hybrid materials (such as cellulose, chitin and lignin) has not been conducted, such novel hydrophilic materials hold promising prospects under the background of green chemistry and sustainable development. This study serves as a preliminary exploration in this emerging research field.

**Figure 2:**
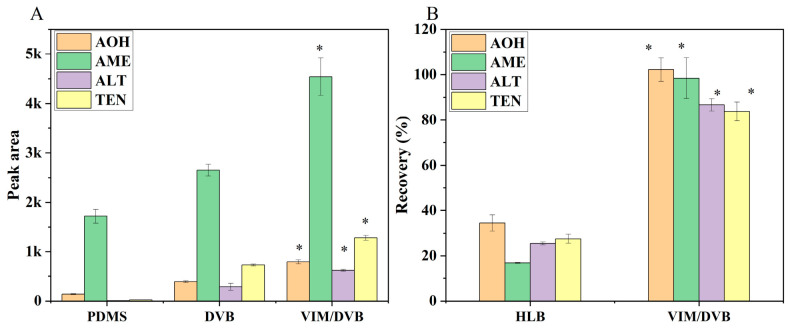
Comparison of extraction efficiencies between the homemade fiber and a commercial polydimethylsiloxane (PDMS) fiber (**A**). Comparison of extraction efficiencies between the solid-phase extraction method using hydrophilic–lipophilic balanced (HLB) column and the homemade solid-phase microextraction method (**B**). AOH: alternariol; AME: alternariol monomethyl ether; ALT: altenuene; TEN: tentoxin. Error bars represent the standard deviation (*n* = 3). * indicates a significantly higher peak area or recovery of the target compound compared with the other methods (*p* < 0.05).

A comparison of extraction efficiencies was conducted between the VIM/DVB-coated fibers, DVB-coated fibers, commercial polydimethylsiloxane (PDMS) fibers, and hydrophilic–lipophilic balanced (HLB) SPE columns under identical conditions. As depicted in Figure 2A, the efficiencies of PDMS and DVB fibers were only 1.7–37.8% and 46.1–58.4% of that of the VIM/DVB fibers, respectively. This inferior performance is likely due to the lack of polar functional groups in PDMS and DVB, which limits their affinity for the relatively polar *Alternaria* toxins [48,49]. In contrast, the superior efficiency of the VIM/DVB coating may be attributed to the electrostatic forces generated by the C-N-bonded p-π conjugated structure of VIM. In addition, the aromatic conjugated structures of the coating can form strong π–π stacking interactions with benzene rings of *Alternaria* toxins. Polar functional groups on the coating surface also serve as potential sites for electrostatic attraction, further improving binding affinity toward target analytes. The synergistic effect of multiple intermolecular interactions endows the SPME fiber with favorable extraction capability. Similarly, the VIM/DVB-SPME method yielded significantly higher recoveries (83.8–102.2%) compared to HLB SPE columns (16.8–34.5%) (Figure 2B). These findings demonstrate that the developed SPME method offers significant advantages over conventional SPE for the analysis of *Alternaria* toxins in edible mushrooms.

To evaluate the practical applicability of the developed SPME–HPLC–MS/MS method, *Pleurotus ostreatus* samples were spiked with standard mixtures of four *Alternaria* toxins at concentrations of 10, 50 and 200 µg/kg. Recovery experiments were performed in triplicate for each spiking level. As shown in Table S4, method recoveries for all *Alternaria* toxins were within 80.6–111.6%, with relative standard deviations ranging from 1.0% to 9.4%. These results demonstrate high accuracy and reproducibility of the method, confirming its suitability for the routine analysis of *Alternaria* toxins in edible mushrooms. However, the method validation in this study was mainly performed using the *Pleurotus ostreatus* matrix. Since the matrix compositions of different edible mushrooms vary slightly, matrix effects may fluctuate to a certain extent. Therefore, cross-species precise quantitative comparison of the quantitative results among different edible mushrooms should be interpreted with caution.

### 3.3. Occurrence and Distribution of Alternaria Toxins in Edible Mushrooms

**Figure 3:**
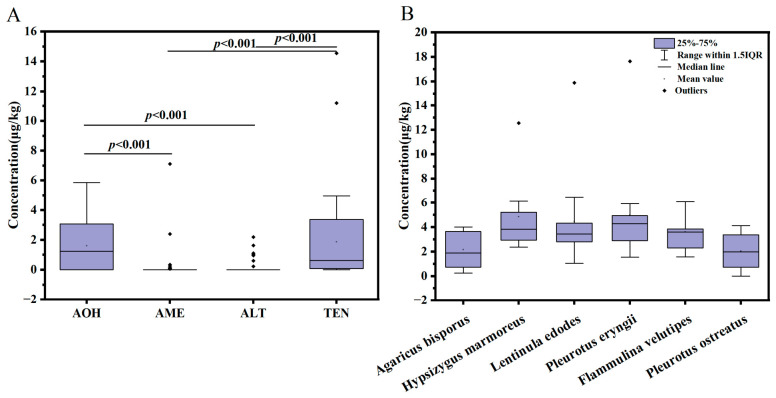
Concentrations of different *Alternaria* toxins in edible mushrooms (**A**) and total *Alternaria* toxin concentrations in different edible mushrooms (**B**). AOH: alternariol; AME: alternariol monomethyl ether; ALT: altenuene; TEN: tentoxin.

The concentrations of the four toxins in edible mushrooms were presented in Figure 3A and Table S5. Notably, all tested mushroom samples contained at least one of these four toxins, indicating widespread contamination. The distribution of different *Alternaria* toxins varied significantly across the samples. TEN exhibited the highest concentration (1.86 ± 2.60 μg/kg, 95% CI: 1.20–2.51 μg/kg) with detection frequency of 76.2%, followed by AOH with a concentration of 1.61 ± 1.80 μg/kg (95% CI: 1.16–2.07 μg/kg) and a detection frequency of 55.6%. In contrast, the concentrations and detection frequencies of alternariol monomethyl ether (AME; 0.17 ± 0.94 μg/kg, 95% CI: 0.00–0.41 μg/kg, 20.6%) and altenuene (ALT; 0.14 ± 0.42 μg/kg, 95% CI: 0.03–0.25 μg/kg, 12.7%) were markedly lower. Statistical analysis revealed that the levels of AOH and TEN were significantly higher than those of AME and ALT (*p* < 0.05), while no significant differences were observed between AOH and TEN, or between AME and ALT.

Although the mean concentrations of *Alternaria* toxins differed among various edible mushrooms, these interspecific variations were not statistically significant (Figure 3B and Table S6). Specifically, *Pleurotus eryngii*, *Hypsizygus marmoreus*, and *Lentinula edodes* exhibited relatively high total *Alternaria* toxin levels, with concentrations of 4.99 ± 4.18 μg/kg, 4.84 ± 3.18 μg/kg, and 4.46 ± 3.55 μg/kg, respectively. This was followed by *Flammulina velutipes* at 3.62 ± 1.60 μg/kg. *Agaricus bisporus* contained the lowest total concentration of *Alternaria* toxins, at 2.16 ± 1.52 μg/kg.

**Figure 4:**
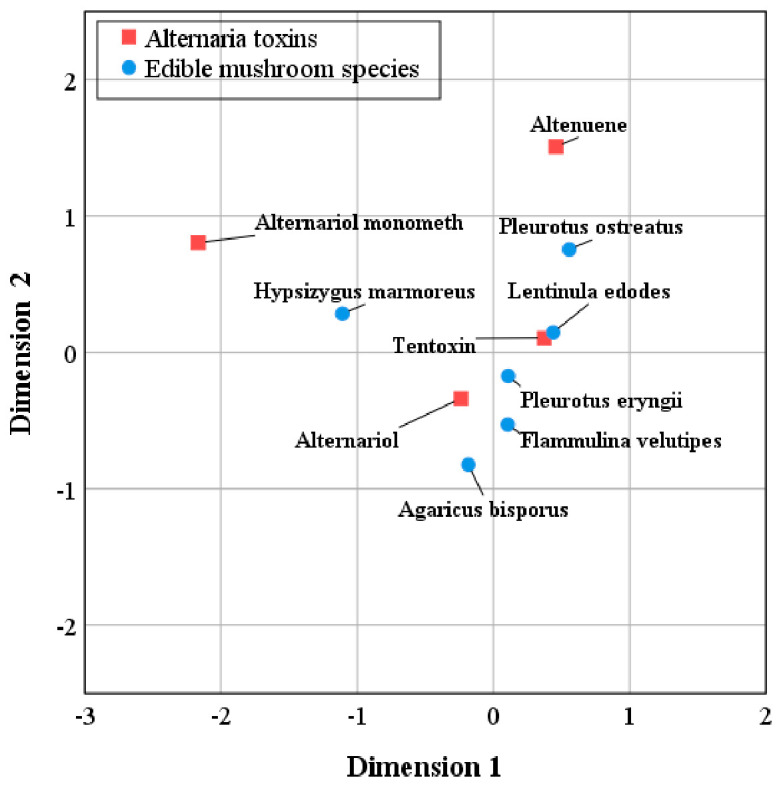
Multivariate correspondence analysis plot between edible mushrooms and *Alternaria* toxins.

To further clarify the association between *Alternaria* toxins and edible mushrooms, a multiple correspondence analysis was performed, with results shown in Figure 4. Dimension 1 and Dimension 2 explained 56.5% and 19.2% of the total variance, respectively. *Lentinula edodes* was prone to TEN, while *Pleurotus eryngii* was even more susceptible to AOH-TEN co-occurrence. *Pleurotus ostreatus* tended to contain ALT and TEN, whereas ALT was the most prevalent toxin in both *Agaricus bisporus* and *Flammulina velutipes*. For *Hypsizygus marmoreus*, no significant bias toward any specific toxin was observed.

Based on the distribution of *Alternaria* toxins in other foods, we speculate that the observed differences in *Alternaria* toxin distribution among different edible mushrooms may be attributed to the combined effects of the host microenvironment, cultivation conditions, toxin stability, and contamination pathways, which collectively modulate the production, accumulation, and persistence of these toxins [6,50]. Species with higher surface area-to-volume ratios (e.g., *Flammulina velutipes* with elongated stipes and small caps) or porous hymenium structures (e.g., Lentinula edodes) may provide more attachment sites and protected microenvironments for Alternaria spores compared to smooth-capped species like Agaricus bisporus [50]. Of note, additional external factors, including the production environment, cultivation mode, harvesting season, and storage/transportation conditions, can also influence contamination levels [18]. Fungi of the genus Alternaria readily grow and proliferate under favorable temperature and humidity, such that variations in sample origin and handling conditions may lead to distinct toxin contamination profiles [5]. Since the present study primarily focused on contamination status and dietary risk assessment, future investigations with an expanded sampling scope and larger sample size are warranted to systematically elucidate how origin and storage conditions shape Alternaria toxin contamination.

### 3.4. Risk Assessment of Alternaria Toxins in Edible Mushrooms

**Figure 5:**
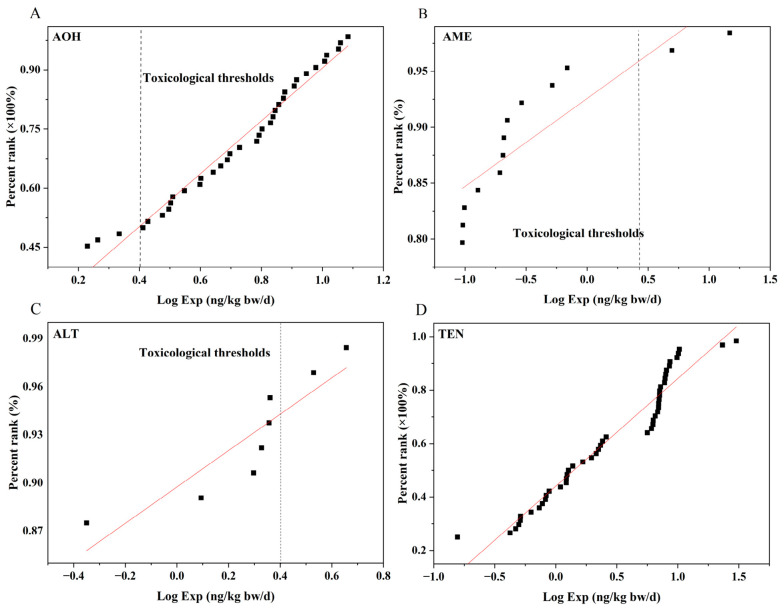
Exposure distribution curves of *Alternaria* toxins in edible mushrooms. (**A**) AOH (alternariol); (**B**) AME (alternariol monomethyl ether); (**C**) ALT (altenuene); (**D**) TEN (tentoxin).

Although the concentrations of *Alternaria* toxins detected in edible mushrooms were generally lower than those reported in cereals, vegetables, and fruits on the EU market, their potential risks cannot be ignored [15]. The results of the dietary exposure risk assessment for *Alternaria* toxins in edible mushrooms are presented in Figure 5. This assessment indicates a considerable probability that the intake of *Alternaria* toxins from edible mushrooms exceeds the toxicological threshold. Among the four toxins, AOH poses the highest dietary exposure risks, with 51% of edible mushrooms exceeding the toxicological threshold (Figure 5A). The dietary exposure risk of AME and ALT is relatively low, accounting for only 4% and 6% of samples, respectively (Figure 5B and Figure 5C). Although TEN exhibits a relatively high detected level, its toxicological threshold is also elevated; thus, the intake of TEN did not reach the toxicological threshold in any of the samples (Figure 5D). In summary, priority should be given to monitoring the contamination status and evaluating the health risks of AOH and ALT in edible mushrooms, followed by attention to the associated risks of AME.

Significant differences in dietary risks were observed among different edible mushrooms (Figure S4). Regarding AOH, except for *Pleurotus ostreatus*, which has an average hazard quotient (HQ) < 1, all other edible mushrooms have HQ values exceeding 1. This indicates that AOH in edible mushrooms poses health risks to humans. Among these species, *Hypsizygus marmoreus* had the highest average HQ value (2.04), followed by *Flammulina velutipes* (1.64), *Pleurotus eryngii* (1.58), *Lentinula edodes* (1.4), and *Agaricus bisporus* (1.04), all of which exceeded the toxicological threshold. Therefore, close attention should be paid to the dietary risks of AOH in edible mushrooms. For AME, the HQ values of all edible mushrooms were an overall low dietary risk. However, the dietary risks of AME in *Agaricus bisporus* and *Pleurotus eryngii* were relatively higher than those in other species, which merits attention. With respect to ALT, although the average HQ value of ALT in *Lentinula edodes* was lower than 1, its 75th percentile HQ value reached 0.5, which is much higher than that in other edible mushrooms. This suggests that increased attention should be directed to the risk of ALT in *Lentinula edodes*. Additionally, despite the relatively high detection rate and concentration of TEN in all edible mushrooms, its HQ value was lower than 0.1, indicating a low relative risk.

The dietary exposure assessment in this study was based on the raw consumption scenario of edible mushrooms, without considering the effects of cooking and processing. Studies have shown that *Alternaria* toxins can be partially degraded or leached during cooking processes such as heating and boiling [51]. Therefore, the real human intake may be lower than the estimated value in this study, rendering the assessment results relatively conservative. In addition, the relevant calculations are based on fresh mushrooms, and the analytical validation primarily focused on *Pleurotus ostreatus*; therefore, comparisons between different species should be treated with great caution. Estimates of dietary exposure to *Alternaria* toxins from the consumption of mushrooms should be interpreted conservatively. Constrained by available research data, the effects of different cooking methods on toxin levels warrant further systematic investigation, and such uncertainties should be taken into account when interpreting the results.

## 4. Conclusions

Overall, this study successfully developed an SPME-HPLC-MS/MS method for the detection of *Alternaria* toxins in edible mushrooms. The established method demonstrates a favorable linear range (1–200 μg/kg), low limits of detection (0.01–0.26 μg/kg) and limits of quantification (0.05–0.86 μg/kg). Experimental results showed that *Alternaria* toxins are widely detected in edible mushrooms collected from Guiyang, with TEN and alternariol AOH as the predominant toxins. *Lentinula edodes* is strongly associated with TEN; *Pleurotus eryngii* is prone to AOH-TEN co-occurrence; ALT is prevalent in *Agaricus bisporus* and *Flammulina velutipes*, while *Pleurotus ostreatus* tends to contain both ALT and TEN. Dietary risk assessment indicated that AOH may present relatively high potential health risks, as 51% of samples exceed the EFSA toxicological threshold. Additionally, the HQ of AOH in five mushroom species (excluding *Pleurotus ostreatus*) exceeds 1. In contrast, AME and ALT present a low potential risk, while no significant risk was observed for TEN. However, these risk estimates are limited by the TTC-based assumption for ALT and restricted matrix validation only using *Pleurotus ostreatus*, which should be carefully interpreted. Considering that toxin levels detected in some samples approached or exceeded existing toxicological thresholds of concern, our findings further demonstrate the necessity of conducting systematic dietary exposure assessments and, based on such evaluations, scientifically determining whether relevant safety standards should be established.

## Figures and Tables

**Table 1:** The detection limits of *Alternaria* toxins in this study and previous studies.

Sample	Analytical Method	Sample Preparation Method	LOD	References
Nutmeg, coix seed and alpinia officinarum rhizoma	UPLC-MS/MS	QuEChERS	0.003–0.45 μg/kg	[9]
Fruit Puree	UPLC-MS/MS	QuEChERS	0.18–0.53 μg/kg	[7]
Wolfberry	UPLC-MS/MS	QuEChERS	0.07–0.24 μg/kg	[19]
Fruits	LC-MS/MS	QuEChERS	0.06–2.00 μg/kg	[36]
Maize and wheat	LC-MS/MS	QuEChERS	0.20 μg/kg	[37]
Chili paste, eggplant, ketchup, pepper and tomato	UHPLC-MS/MS	SPE	0.05–2 μg/kg	[17]
Rice, sesame, tomato and apple juice	LC-MS/MS	SPE	0.04–1.67 μg/kg	[38]
Drinking water	UPLC-MS/MS	SPE	0.005–0.05 ng/kg	[39]
Barley	LC-MS/MS	SPE	0.05–2.5 μg/kg	[40]
Cherries and oranges	Immunochromatographic strip	SPE	10 μg/kg	[41]
Cherries and oranges	Colorimetric immunosensor	-	0.16 μg/kg	[42]
Olive oil	UHPLC-MS/MS	LLME	0.05–0.5 μg/kg	[29]
Human urine	LC-MS/MS	LLE	0.001–0.06 μg/kg	[43]
Juice, flour and tomato ketchup	OS-ELISA	-	0.08 μg/kg	[44]
Fruit juice, maize and flour	icCLEIA	-	0.068 μg/kg	[45]
Fruit juice	icCLEIA	-	0.2 μg/kg	[46]
Wheat	icELISA	-	0.7–1.0 μg/kg	[47]
Cornflakes	HPLC–UV/DAD	SPME	25 ± 6 μg/kg	[35]
Ice wine	LC-DAD	SPME	70 μg/kg	[34]
Edible mushrooms	HPLC-MS/MS	SPME	0.01–0.26 μg/kg	This work

HPLC: high-performance liquid chromatography. UHPLC: ultrahigh-performance liquid chromatography-tandem mass spectrometry. OS-ELISA: open sandwich enzyme-linked immunosorbent assay. icCLEIA: indirect competitive chemiluminescence enzyme immunoassay. UV/DAD: diode array UV detection. QuEChERS: quick, easy, cheap, effective, rugged, and safe method. SPE: solid-phase extraction. LLME: liquid–liquid microextraction. LLE: liquid–liquid extraction. SPME: solid-phase microextraction.

## Data Availability

The original contributions presented in this study are included in the article/Supplementary Materials. Further inquiries can be directed to the corresponding authors.

## Supplementary Materials

The following supporting information can be downloaded at: https://www.mdpi.com/article/10.3390/foods15111992/s1.
