# Supplementary material for: Occurrence and Dietary Risk Assessment of Alternaria Toxin in Edible Mushrooms: An Application of a Hydrophilic Solid-Phase Microextraction Fiber
Source: Foods. 2026 Jun 3;15(11):1992. doi: 10.3390/foods15111992 (PMC13256904; doi:10.3390/foods15111992)
Supplement: Supplementary file 1 [file foods-15-01992-s001.zip › foods-4324858-supplementary.pdf]

Supplementary Information

Table S1. Retention time and mass spectrometric parameters of 4 *Alternaria* toxins

Component	Molecular structure	Ionization mode	Precursor Ion (m/z)	Product Ion (m/z)	Dwell (s)	Fragmentor (V)	Collision Energy (V)
AOH	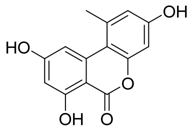	ESI$^-$	257.0	147.2<br>213.2*	50	120	42<br>32
AME		ESI$^-$	271.2	228.2<br>256.2*	50	100	42<br>28
ALT	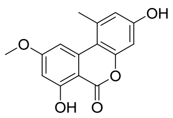	ESI$^-$	290.9	185.9<br>214.1*	50	70	36<br>22
TEN	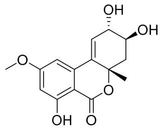	ESI$^-$	413.4	140.8*<br>271.1	50	120	23<br>27

The method settings are as follows: Ionization method: Electrospray Ionization (ESI) source; Scanning mode: Negative Ion Mode; Monitoring modality: Multi-Response Monitoring (MRM); Capillary current: 4023 nA; Nebulizer pressure: 35.0 psi; Dry gas temperature: 300 ℃; Drying gas flow: 8.0 L/min; Sheath gas temperature: 250 ℃; Sheath gas flow: 10.0 L/min.

*: the quantification ion

AOH: alternariol

AME: alternariol monomethyl ether

ALT: altenuene

TEN: tentoxin

Table S2. The structural properties of SPME fiber materials

Material	Surface area (m^2/g)	Pore volume (cm^3/g)	Pore diameter (nm)
1-vinylimidazole /divinylbenzene	121.3	0.43	3.8
divinylbenzene	4.6	0.008	3.1

Table S3. Liner range and matrix effect of four *Alternaria* toxins (n=3)

Component	Liner range [a] (μg/kg)	Regression equation	Correlation coefficient	LOD (μg/kg)	LOQ (μg/kg)	Matrix effect （%）	RSD (%)
							Fiber-to-fiber
Alternariol	1–200	y=20.412x+4.0301	0.998	0.26	0.86	-78.70	5.02
Alternariol monomethyl	1–200	y=440.07x+604.91	0.999	0.01	0.05	-78.19	2.99

ether							
Altenuene	1–200	y=14.638x+56.565	0.997	0.21	0.69	-50.63	2.91
Tentoxin	1–200	y=20.721x+59.164	0.997	0.08	0.25	-83.83	8.96

LOD and LOQ are calculated based on target peak areas corresponding to signal-to-noise ratios of S/N=3 and S/N=10, respectively. [a] Linearity was evaluated by constructing matrix-matched calibration curves in Pleurotus ostreatus extracts.

The matrix effect was evaluated by comparing the calibration curves derived from the calibration ranges in solvent and matrix.

Table S4. Recovery and repeatability of the method for the analysis of *Alternaria* toxins in *Pleurotus ostreatus*

Component	Spiked (μg/kg)	Recovery (%)	RSD (%)
AOH	10	93.9 ± 1.2	1.3
	50	80.6 ± 4.1	5.1
	200	107.2 ± 7.2	6.7
AME	10	82.7 ± 0.9	1.1
	50	90.3 ± 2.9	3.2
	200	94.0 ± 5.8	6.1
ALT	10	107.0 ± 6.0	5.6
	50	107.1 ± 3.3	3.1
	200	100.8 ± 3.5	3.4
TEN	10	90.9 ± 2.4	2.6
	50	111.6 ± 10.5	9.4
	200	101.0 ± 1.1	1.0

AOH: alternariol； AME: alternariol monomethyl ether ； ALT: altenuene ； TEN: tentoxin

Table S5 Concentrations of different *Alternaria* toxins in edible mushrooms

Component	Concentration (μg/kg)					95% confidence intervals for the mean (μg/kg)	Detection rate (%)
	Mean	Median	Standard deviation	Maximum	Minimum		
AOH	1.61	1.24	1.80	5.85	ND	1.16-2.07	55.6
AME	0.17	ND	0.94	7.11	ND	0.00-0.41	20.6
ALT	0.14	ND	0.42	2.19	ND	0.03-0.25	12.7
TEN	1.86	0.61	2.60	14.55	ND	1.20-2.51	76.2
∑toxin[a]	3.78	3.36	3.07	17.63	ND	3.00-4.55	98.4

ND: not detected

AOH: alternariol

AME: alternariol monomethyl ether

ALT: altenuene

TEN: tentoxin

[a]: the sum of the concentrations of AOH, AME, ALT and TEN in edible mushrooms is denoted by ∑toxin, indicating the presence of at least one toxin.

Table S6 Total *Alternaria* toxins concentrations in different edible mushrooms

Sample categories	Number of samples	Concentration (μg/kg)					Detection Rate (%)
		Mean	Median	Standard deviation	Maximum	Minimum	
Agaricus bisporus	7	2.16	1.87	1.52	4.01	0.25	100
Hypsizygus marmoreus	9	4.84	3.82	3.18	12.56	2.35	100
Lentinula edodes	14	4.46	3.43	3.55	15.87	1.04	100
Pleurotus eryngii	12	4.99	4.28	4.18	17.63	1.53	100
Flammulina velutipes	9	3.62	3.58	1.60	6.08	1.56	100
Pleurotus ostreatus	12	2.05	1.98	1.45	4.12	ND	91.7

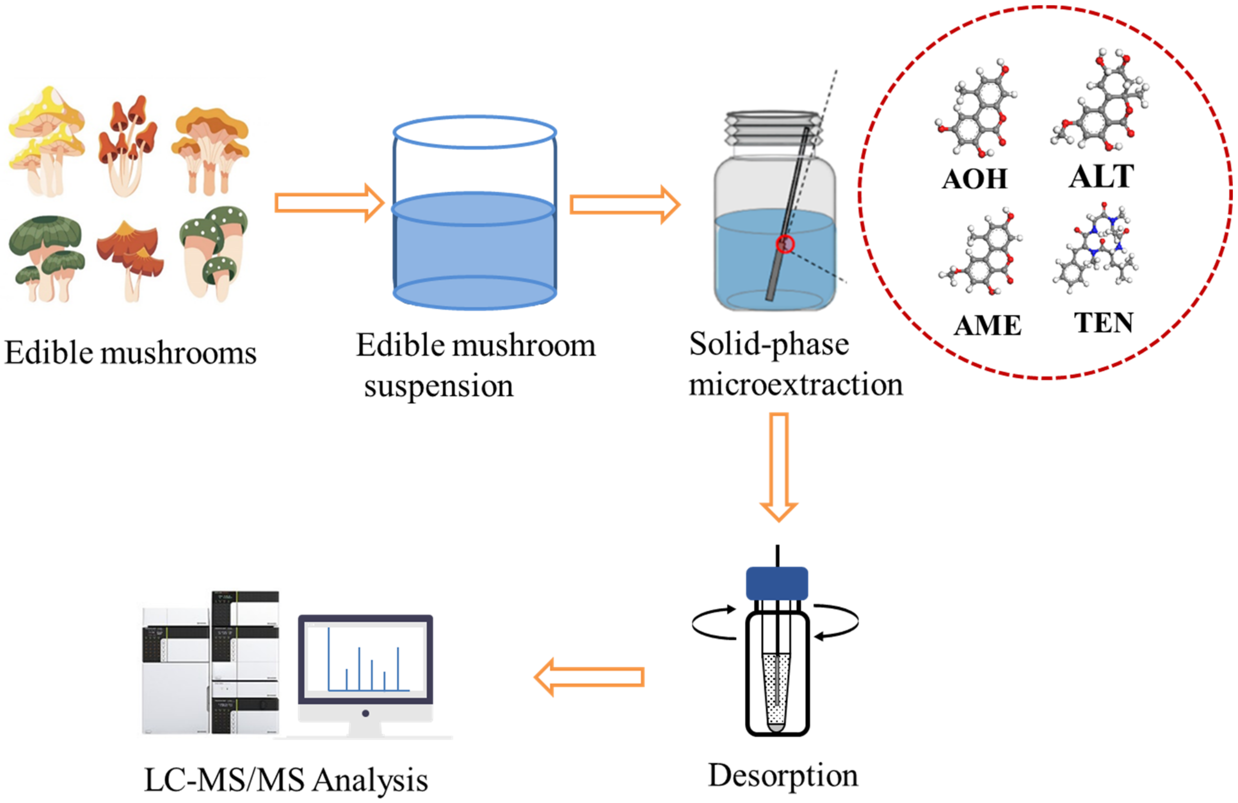

Figure S1. Schematic diagram of SPME workflow

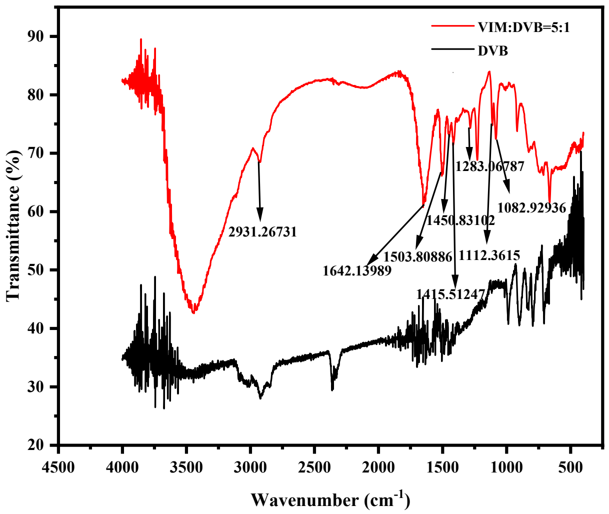

Figure S2. Fourier-transform infrared (FTIR) spectrum of synthetic adsorbents.

VIM: 1-vinylimidazole, DVB: divinylbenzene.

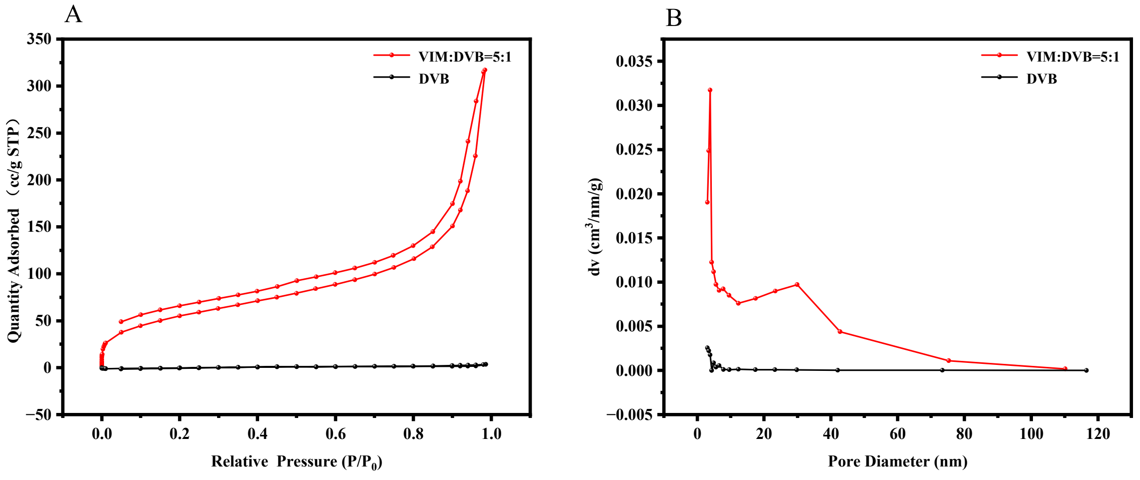

Figure S3. The N_2 adsorption-desorption isotherms (A) and pore size distribution curves (B) for synthetic adsorbents. VIM: 1-vinylimidazole, DVB: divinylbenzene.

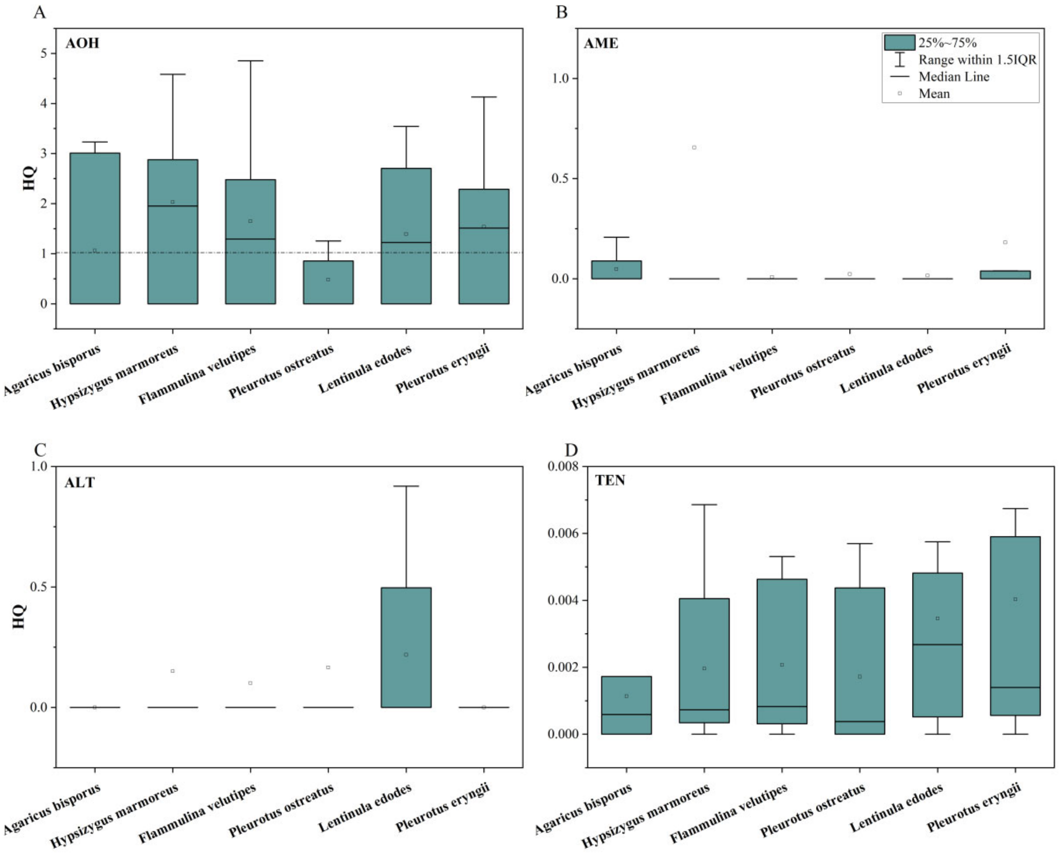

Figure S4. Comparison of hazard quotients for *Alternaria* toxins in edible mushrooms. AOH: alternariol, AME: alternariol monomethyl ether, ALT: altenuene, TEN: tentoxin.
